# LpxT-Dependent Phosphorylation of Lipid A in *Escherichia coli* Increases Resistance to Deoxycholate and Enhances Gut Colonization

**DOI:** 10.3389/fmicb.2021.676596

**Published:** 2021-05-04

**Authors:** Xudong Tian, Guillaume Manat, Elise Gasiorowski, Rodolphe Auger, Samia Hicham, Dominique Mengin-Lecreulx, Ivo Gomperts Boneca, Thierry Touzé

**Affiliations:** ^1^Université Paris-Saclay, CEA, CNRS, Institute for Integrative Biology of the Cell (I2BC), Gif-sur-Yvette, France; ^2^Institut Pasteur, Unité Biologie et Génétique de la Paroi Bactérienne, Paris, France; ^3^Université de Paris, Sorbonne Paris Cité, Paris, France

**Keywords:** lipopolysaccharides, lipid A, two-component system, antibiotic resistance, polymyxin B, bile acid

## Abstract

The cell surface of Gram-negative bacteria usually exhibits a net negative charge mostly conferred by lipopolysaccharides (LPS). This property sensitizes bacterial cells to cationic antimicrobial peptides, such as polymyxin B, by favoring their binding to the cell surface. Gram-negative bacteria can modify their surface to counteract these compounds such as the decoration of their LPS by positively charged groups. For example, in *Escherichia coli* and *Salmonella*, EptA and ArnT add amine-containing groups to the lipid A moiety. In contrast, LpxT enhances the net negative charge by catalyzing the synthesis of tri-phosphorylated lipid A, whose function is yet unknown. Here, we report that *E. coli* has the intrinsic ability to resist polymyxin B upon the simultaneous activation of the two component regulatory systems PhoPQ and PmrAB by intricate environmental cues. Among many LPS modifications, only EptA- and ArnT-dependent decorations were required for polymyxin B resistance. Conversely, the acquisition of polymyxin B resistance compromised the innate resistance of *E. coli* to deoxycholate, a major component of bile. The inhibition of LpxT by PmrR, under PmrAB-inducing conditions, specifically accounted for the acquired susceptibility to deoxycholate. We also report that the kinetics of intestinal colonization by the *E. coli lpxT* mutant was impaired as compared to wild-type in a mouse model of infection and that *lpxT* was upregulated at the temperature of the host. Together, these findings highlight an important function of LpxT and suggest that a tight equilibrium between EptA- and LpxT-dependent decorations, which occur at the same position of lipid A, is critical for the life style of *E. coli*.

## Introduction

**FIGURE 1:**
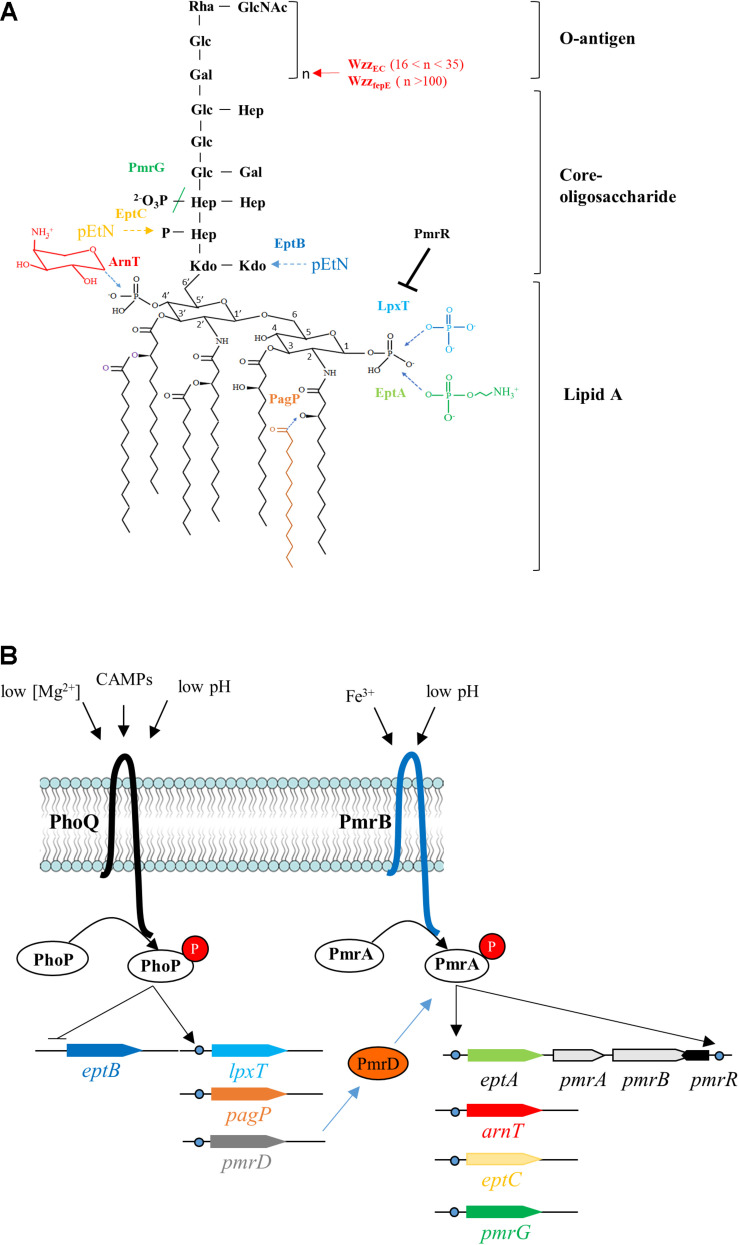
Lipopolysaccharide modifications and their regulation in *E. coli*. **(A)** The structure of the unmodified lipopolysaccharide (LPS) from *E. coli* is shown in black. The chemical modifications and/or the corresponding enzymes catalyzing them are color-coded. The added chemical groups and the enzymes are similarly colored and the dotted arrows indicate the sites of modification. The colored slash indicates the hydrolysis of a chemical bond by PmrG. Wzz_*EC*_ and Wzz_fepE_ control the number of O-antigen subunits (n) to be added to the lipid A–core, with the corresponding value of n indicated between brackets. PmrR is a membrane peptide which inhibits the phosphotransferase activity of LpxT. **(B)** The two-component systems (TCS) PhoPQ and PmrAB regulate the expression of genes encoding the major LPS modifying enzymes, the regulatory membrane peptide PmrR, the TCS connector PmrD or PmrAB proteins. The stimuli that are recognized by the sensor proteins from these TCS are indicated. Rha, rhamnose; GlcNAc, *N*-acetylglucosamine; Glc, glucose; Hep, heptose; Gal, galactose; Kdo, 3-deoxy-D-manno-oct-2-ulosonic acid; pEtN, phosphoethanolamine.

The lipopolysaccharide (LPS) constitutes the outermost component of Gram-negative bacteria preventing the entry of a wide variety of noxious compounds such as antibiotics and detergents (Nikaido, 2003). The LPS is composed of a lipid A moiety, which anchors the LPS to the outer membrane, a core oligosaccharide and an O-antigen polymer (Figure 1A). The lipid A is primarily synthesized as a β-(1′,6)-linked disaccharide of glucosamine, which is hexaacylated and flanked by phosphate groups at positions 1 and 4′ (Figure 1A). The lipid A can undergo different chemical modifications in response to environmental cues (Raetz and Whitfield, 2002; Raetz et al., 2007; Simpson and Trent, 2019). In many species, among major pathogens like *Escherichia coli*, *Salmonella*, *Yersinia pestis*, *Pseudomonas aeruginosa*, and *Klebsiella pneumoniae*, a significant portion of lipid A is constitutively phosphorylated at position 1, yielding lipid A 1-diphosphate species (lipid A 1-PP) (Figure 1A; Touzé et al., 2008; Nowicki et al., 2014). In *E. coli* cells grown in rich nutrient broth, about one third of the total lipid A presents a diphosphate group at position 1 (Touzé et al., 2008). This modification is catalyzed, at the periplasmic side of the plasma membrane, by LpxT, which transfers a phosphate group from undecaprenyl pyrophosphate (C_55_-PP) to lipid A releasing undecaprenyl phosphate (C_55_-P) as a by-product (Touzé et al., 2008). C_55_-P constitutes the lipid carrier for translocation of subunits of cell-wall polysaccharides (e.g., peptidoglycan, O-antigen) across the plasma membrane. It is released as C_55_-PP during polymers biogenesis on the outer side of the plasma membrane, where LpxT uses it as a substrate and thus contributes to its essential recycling in a redundant manner with other C_55_-PP phosphatases (El Ghachi et al., 2005; Manat et al., 2014).

In *E. coli* and *Salmonella*, the PmrAB (also referred as BasRS) two-component regulatory system (TCS) controls most of the LPS modifications (Figures 1A,B). The environmental cues, which are perceived by the sensor PmrB, are mildly acidic pH (Soncini and Groisman, 1996) and high concentrations of Fe^3+^ (Hagiwara et al., 2004). Under these conditions, PmrB phosphorylates the response regulator PmrA and conversely, when the signal is absent, PmrB dephosphorylates PmrA. Under PmrAB-inducing conditions, the peptide PmrR inhibits the LpxT-dependent modification (Herrera et al., 2010; Kato et al., 2012), while EptA and ArnT add phosphoethanolamine (pEtN) and 4-amino-4-deoxy-L-arabinose (L-Ara4N) groups onto the phosphate groups at positions 1 and 4′ of lipid A, respectively (Figures 1A,B; Trent et al., 2001; Lee et al., 2004). EptA and LpxT operate their specific decoration at the same position of lipid A, i.e., the phosphate group at position 1, in a competitive manner (Herrera et al., 2010; Kato et al., 2012). The addition of positively charged groups decreases the overall negative charge at the cell surface conferring higher resistance to iron and cationic antimicrobial peptides (CAMPs) such as polymyxin B (Zhou et al., 2001). On the core oligosaccharide, PmrG dephosphorylates Heptose II, while EptB and EptC add pEtN on 3-deoxy-D-manno-oct-2-ulosonic acid (Kdo) and Heptose I, respectively (Figure 1A; Reynolds et al., 2005; Tamayo et al., 2005; Nishino et al., 2006). In *Salmonella*, the modality of the O-antigen polymer synthesis is controlled by PmrA-upregulated Wzz_ST_ and Wzz_fepE_, which promote the synthesis of long and very long O-antigens, respectively, thereby contributing to CAMPs resistance (Delgado et al., 2006; Pescaretti et al., 2011; Chen and Groisman, 2013). In *Salmonella*, PmrAB is also indirectly activated via another TCS, PhoPQ, which is stimulated under low Mg^2+^ concentrations, mildly acidic pH and by CAMPs (García Véscovi et al., 1996; Bader et al., 2005; Prost et al., 2007). The PhoQ regulator induces the production of PmrD protein, which prevents PmrA dephosphorylation by PmrB, thus maintaining PmrA in its active conformation (Figure 1B; Kox et al., 2000). In *E. coli*, the PmrD connector was first described as non-functional (Winfield and Groisman, 2004), but another report showed that PmrD stimulated PmrA-dependent L-Ara4N and pEtN modifications under low Mg^2+^ conditions (Rubin et al., 2015). LpxT was also found to be upregulated by PhoPQ under low Mg^2+^ conditions in *Salmonella enterica* and *E. coli* (Hong et al., 2018). PagP, which is also upregulated by PhoPQ, adds a palmitate to lipid A (Bishop, 2005), but this modification was not observed in *E. coli* under mildly acidic conditions in contrast to *Salmonella* (Zhou et al., 1999; Gibbons et al., 2005). Another signal like envelope stress is apparently required to elicit this modification in *E. coli* (Eguchi et al., 2004). The PagP-dependent palmitoylation promotes CAMPs resistance in *Acinetobacter baumannii* by increasing van der Waals interactions within the outer membrane (Boll et al., 2015). Isolates of *E. coli* K-12 exhibiting a constitutive resistance to polymyxin B display mutations within PmrA: a G_53_V mutation in DW137 mutant (Froelich et al., 2006), and G_53_E/A_42_T mutations in WD101 mutant (Trent et al., 2001). In these mutants, PmrA is locked in its active conformation because PmrB less efficiently dephosphorylates it. Consequently, the modifications with L-Ara4N and pEtN are constitutive and lipid A 1-PP species are no longer observed (Touzé et al., 2008). Interestingly, DW137 displays increased susceptibility to deoxycholate, a component of the bile to which *E. coli* normally exhibits innate resistance (Froelich et al., 2006). The constitutive stimulation of PmrA also impairs the growth of *Salmonella* in the presence of bile. The expression of Wzz_ST_, which supports the synthesis of abundant long O-antigen chains, was found to be responsible for bile acids susceptibility, while the modifications by L-Ara4N and pEtN had no effect (May and Groisman, 2013). *E. coli* K-12 does not produce O-antigen suggesting that different PmrA-controlled events sensitize *E. coli* and *Salmonella* to bile acids.

In this study, the goal was to determine the external stimuli and the LPS decorations that affect the susceptibility of *E. coli* to polymyxin B and deoxycholate, i.e., two antibacterial agents with opposite charges (cationic *versus* anionic), which are very common in the niches of enterobacteriaceae. We showed that activation of both PhoPQ and PmrAB TCS by different environmental cues increases polymyxin B resistance in *E. coli*, which comes at the expense of loss of innate resistance to deoxycholate. We then provide evidence that bile susceptibility arises from PmrR-dependent inhibition of LpxT. This study highlights the importance of the status of lipid A at position 1 (i.e., phosphate *versus* pEtN modification) for *E. coli* to cope with major antibacterial agents encountered in the gut of mammals.

## Results

### Intricate Stimuli Induce Innate Polymyxin B Resistance in *E. coli*

**FIGURE 2:**
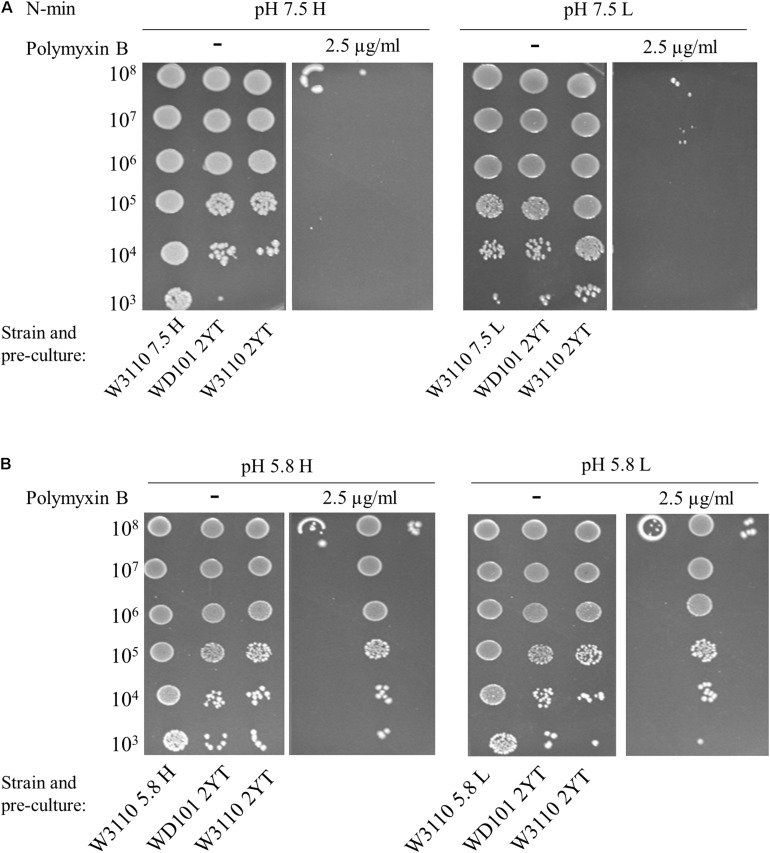
Low Mg^2+^ and low pH do not elicit polymyxin B resistance. W3110 and WD101 stains were grown in 2YT medium or minimal medium (N-min) at pH 7.5 **(A)** or 5.8 **(B)** with 1 mM (H = high, left) or 10 μM (L = low, right) MgCl_2_. 5-μl spots of dilutions of these overnight pre-cultures were then deposited on plates supplemented or not with 2.5 μg/ml of polymyxin B as follows: **(A)** N-min at pH 7.5 with 1 mM (H) or 10 μM (L) MgCl_2_. **(B)** N-min at pH 5.8 with 1 mM (H) or 10 μM (L) MgCl_2_. Approximate numbers of CFU/ml of the loaded suspension, determined according to the OD_600__*nm*_ of the pre-culture, are indicated on the left. The data are representative of at least three independent experiments.

To investigate how PmrAB and PhoPQ respond in *E. coli*, we searched whether it was possible to induce polymyxin B resistance of *E. coli* W3110 strain (WT) by testing different growth conditions. Previous studies have examined the susceptibility of *E. coli* to polymyxin B by measuring survival of cells after 1 h of exposure to 2.5 μg/ml of this compound. In contrast, we here searched for conditions under which *E. coli* cell grew normally in a milieu containing 2.5 μg/ml of polymyxin B. Different stimuli were tested in the challenging milieu (i.e., containing polymyxin B) as well as in the pre-culture (referred as conditioning medium): two pH values (neutral, 7.5 and mildly acidic, 5.8), two concentrations of Mg^2+^ (10 μM, low and 1 mM, high) and the presence or absence of 300 μM FeSO_4_. Fe^2+^ is quickly oxidized to Fe^3+^ serving as a specific signal for activation of the PmrA–PmrB system (Wösten et al., 2000). In minimal medium (N-min) at pH 7.5, both the WT and the PmrA-constitutive WD101 strain were susceptible to polymyxin B, irrespective of the concentration of Mg^2+^ (Figure 2A). At pH 5.8, WD101 became polymyxin B resistant irrespective of the Mg^2+^ concentration, but the WT remained sensitive (Figure 2B). On one hand, WD101 still required an external stimulus (e.g., mildly acidic pH) to express polymyxin B resistance. On the other hand, low pH combined with low Mg^2+^ did not elicit polymyxin B resistance of WT.

**FIGURE 3:**
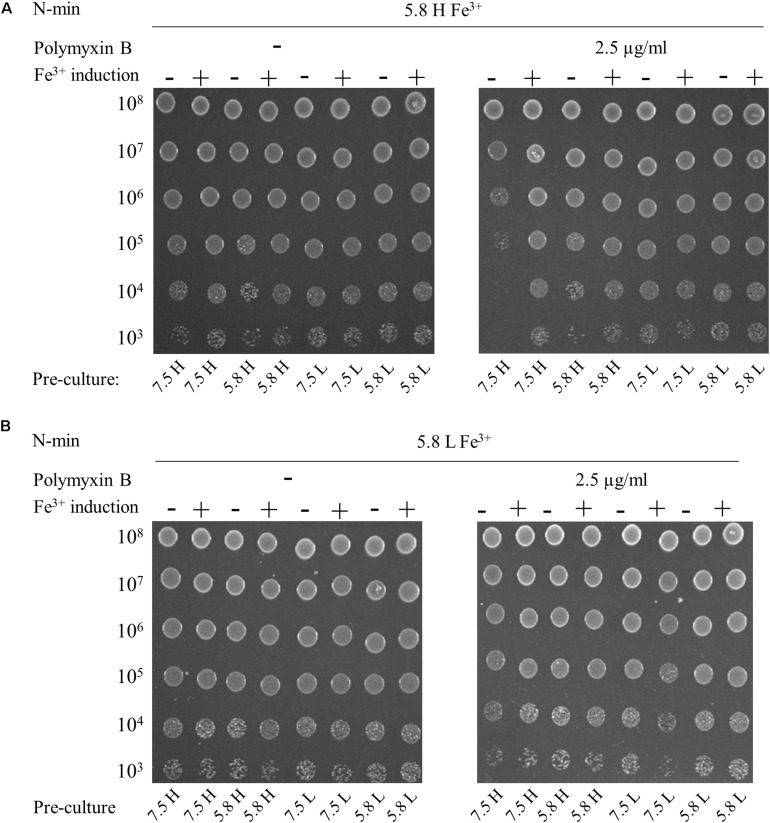
Multiple signals are required to elicit polymyxin B resistance. W3110 strain was grown overnight in N-min at pH 7.5 or 5.8, with 1 mM (H) or 10 μM (L) MgCl_2_. Cells were diluted (1:50) and incubated for 4 h at 37°C in the same medium (–) or in a Fe^3+^ induction medium (+) that is N-min 5.8, high Mg^2+^ and 300 μM Fe^3+^. 5-μl spots of appropriate dilutions of the suspensions were then deposited on plates supplemented or not with 2.5 μg/ml of polymyxin B, as follows: **(A)** N-min at pH 5.8, high Mg^2+^ and 300 μM Fe^3+^ (5.8 H Fe^3+^). **(B)** N-min at pH 5.8, low Mg^2+^ and 300 μM Fe^3+^ (5.8 L Fe^3+^). The data are representative of at least three independent experiments.

The addition of 300 μM Fe^3+^ at pH 7.5 and low or high Mg^2+^ did not elicit polymyxin B resistance of WT (data not shown). In contrast, WT became resistant at pH 5.8, with high Mg^2+^ and high Fe^3+^ irrespective of the conditioning medium, except for a combination of pH 7.5 and high Mg^2+^ (Figure 3A). These results indicate that the presence of stimuli in the conditioning medium was also determinant and that low pH or low Mg^2+^ are both appropriate to trigger resistance of WT. We showed that an additional 4-h induction at pH 5.8, high Mg^2+^ and high Fe^3+^ prior to antibiotic exposure induced resistance after a pre-culture at pH 7.5 and high Mg^2+^ (Figure 3A). When plated at pH 5.8, low Mg^2+^ and high Fe^3+^, WT displayed polymyxin B resistance, irrespective of conditioning medium (Figure 3B).

In conclusion, *E. coli* must be conditioned at least in a milieu of low Mg^2+^ or low pH to grow in presence of polymyxin B in a milieu of low pH and high Fe^3+^. Furthermore, *E. coli* cells grew in the presence of polymyxin B at low pH, low Mg^2+^ and high Fe^3+^, regardless of conditioning.

### PhoPQ Activation Is Required for Polymyxin B Resistance

**FIGURE 4:**
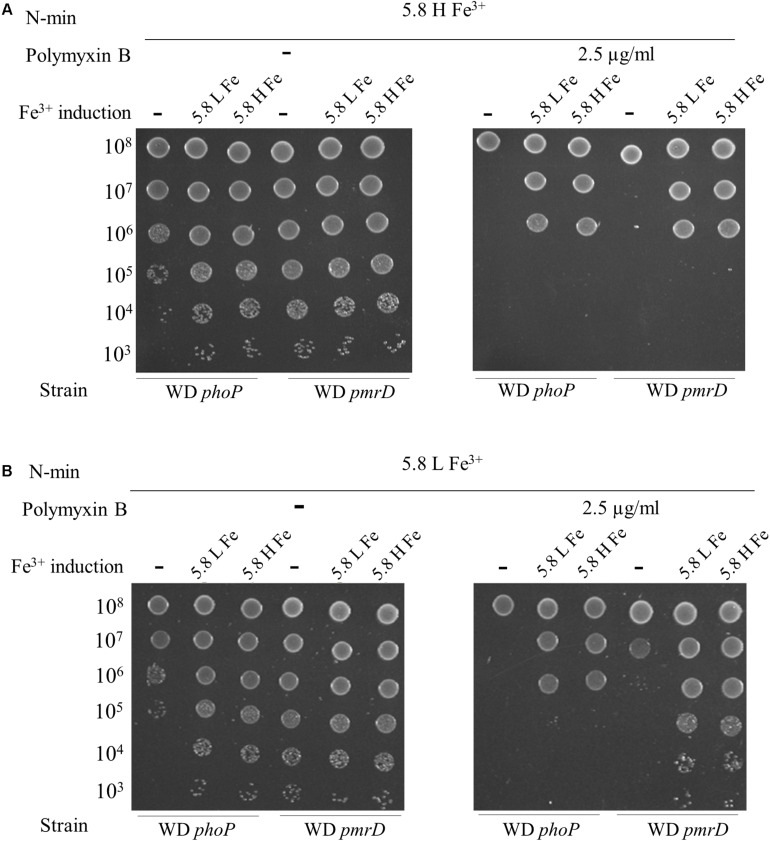
PhoP and PmrD contribute differentially to polymyxin B resistance. W3110 *phoP* and *pmrD* mutants were grown overnight in N-min at pH 7.5 and low Mg^2+^. Cells were diluted (1:50) and incubated for 4 h at 37°C in the same medium (–) or in a Fe^3+^ induction medium (+) that is N-min at pH 5.8, low Mg^2+^ and high Fe^3+^ (5.8 L Fe^3+^) or N-min at pH 5.8, high Mg^2+^ and high Fe^3+^ (5.8 H Fe^3+^). 5-μl spots of appropriate dilutions were then plotted on plates supplemented or not with 2.5 μg/ml of polymyxin B, as follows: **(A)** N-min at pH 5.8, high Mg^2+^ and high Fe^3+^ (5.8 H Fe^3+^). **(B)** N-min at pH 5.8, low Mg^2+^ and high Fe^3+^ (5.8 L Fe^3+^). The data are representative of at least three independent experiments.

The requirement for multiple stimuli to elicit polymyxin B resistance suggested that both PmrAB and PhoPQ were involved. That was expected for PmrAB, while PhoPQ was thought to be only required in the absence of a direct activation of PmrB. We then assessed the role of PhoP and PmrD connector in polymyxin B resistance. The *phoP* and *pmrD* mutants were grown in N-min at pH 7.5 and low Mg^2+^, which provided a good conditioning of WT before exposure to polymyxin B at pH 5.8, high Mg^2+^ and high Fe^3+^ (Figure 3A). In contrast to WT, *phoP* and *pmrD* mutants were polymyxin B susceptible in these conditions (Figure 4A). These data showed that PhoPQ TCS contributes to polymyxin B resistance under these conditions, at least in part via PmrD. A 4 h-induction at pH 5.8, high Fe^3+^, and low or high Mg^2+^ before challenging, restored only partially the resistance of both strains (a decrease of 3 log units in viable counts with respect to WT) (Figure 4A). This implies that the direct activation of PmrAB by low pH and high Fe^3+^ during this 4 h-period was not sufficient in the absence of PmrD or PhoP, even though a partial *phoP-* and *pmrD*-independent resistance occurred.

When plated at pH 5.8, low Mg^2+^ and high Fe^3+^, the *phoP* and *pmrD* mutants still remained polymyxin B susceptible (Figure 4B). The *phoP* mutant displayed a partial resistance after 4 h-induction in the presence of high Fe^3+^, while *pmrD* mutant became fully resistant (Figure 4B). PhoP is required to obtain the full resistance in a PmrD-independent way; hence, the control of gene(s) of the PhoPQ regulon distinct from the PmrAB regulon is likely required. In contrast, the PmrD connector is dispensable as long as the cells are conditioned in low pH and high Fe^3+^ before exposure, which activates both TCS directly.

### PhoP Is Required for Deoxycholate Resistance and Enhances *lpxT* Expression in Rich Medium

**FIGURE 5:**
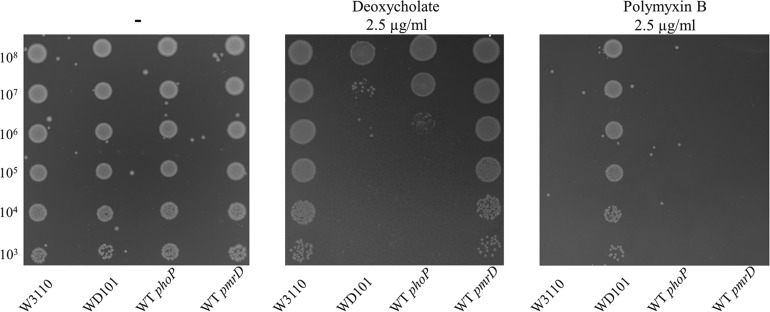
*phoP* is essential for deoxycholate resistance. W3110, WD101, WT *phoP*, and WT *pmrD* strains were grown overnight in 2YT medium. 5-μl spots of serial dilutions of the pre-cultures were then deposited on 2YT-agar plates in the presence or the absence of 2.5 mg/ml of deoxycholate or 2.5 μg/ml of polymyxin B. The data are representative of at least three independent experiments.

**FIGURE 6:**
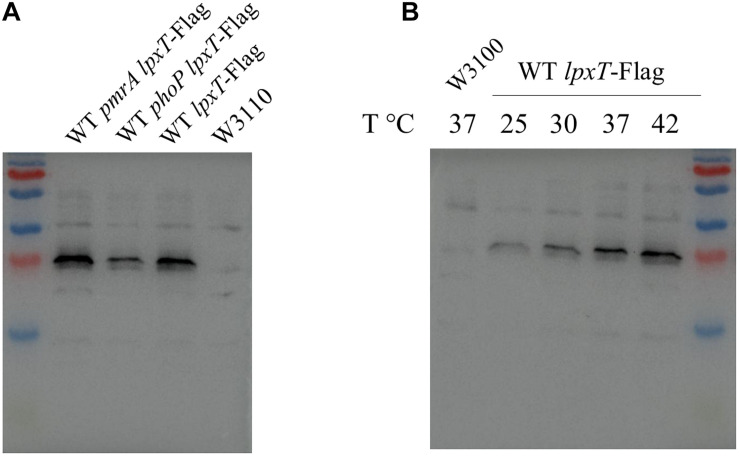
LpxT is upregulated by PhoP and rising temperatures. The 3 × Flag tag-encoding sequence was inserted at the 3′ end of the chromosomal copy of *lpxT* gene in W3110, WT *pmrA* and WT *phoP* strains. **(A)** The three *lpxT*-flag strains and W3110 were grown in 2YT medium at 37°C up to OD_600__*nm*_ = 0.7. Whole protein extracts were prepared and equal amounts of proteins according to the OD_600__*nm*_ of the culture were analyzed by Western blotting. **(B)** WT *lpxT*-flag and W3110 strains were grown in 2YT medium at 25°C before being diluted in fresh 2YT medium at OD_600__*nm*_ = 0.2 and then further incubated at 25, 30, 37, and 42°C for 1 h. Whole protein extracts were prepared and equal amounts of proteins according to the OD_600__*nm*_ of the culture were analyzed by Western blotting. The presented results are representative of three independent experiments.

We next examined the role of PhoPQ and PmrAB on deoxycholate susceptibility. We monitored the growth of *E. coli* strains in 2YT-broth containing 2.5 mg/ml of deoxycholate knowing that the MIC exceeds 100 mg/ml for WT. In contrast to WT and *pmrD* strains, the *phoP* and WD101 strains were susceptible to deoxycholate (Figure 5). In the same conditions, WT, *phoP* and *pmrD* strains were sensitive to polymyxin B, while WD101 was resistant (Figure 5). This finding demonstrates that PhoP is also essential in conferring innate resistance to deoxycholate. PhoPQ was reported to enhance *lpxT* expression by three fold under low Mg^2+^ conditions in *E. coli* (Hong et al., 2018). We then examined whether *lpxT* expression was also upregulated by PhoP when cells were grown on 2YT medium by monitoring the level of 3 × Flag-tagged LpxT. The amount of LpxT protein was similar in WT and *pmrA* strains, while it was reduced by two fold in the *phoP* mutant (Figure 6A) indicating that PhoP also significantly enhances *lpxT* expression in rich nutrient broth.

### *E. coli* Exhibits Deoxycholate Susceptibility in PmrAB-Inducing Conditions

Since the WD101 strain displayed deoxycholate susceptibility, we then questioned whether the WT strain also exhibited deoxycholate susceptibility under PmrAB-inducing conditions. It was not possible to monitor the bacterial growth in the presence of deoxycholate in N-min at pH 5.8 (i.e., required for PmrAB induction) because deoxycholate precipitates at this pH. Therefore, we monitored the survival of the cells after exposure for 1 h to 10 mg/ml of deoxycholate, a dose relevant to the concentration of bile acids in the intestinal tract. The cells were grown in 2YT or in N-min at pH 5.8, low-Mg^2+^ and further conditioned in N-min pH 5.8, low-Mg^2+^ and high Fe^3+^ during 4 h. Then, the cells were challenged or not (control) with deoxycholate in PBS buffer and the surviving cells were numerated. The rate of survival to deoxycholate as compared to the control were 111 ± 25% when cells were grown in 2YT-broth and 39 ± 5% when cells undergo PmrAB-inducing conditions. These data demonstrated that LPS modifications or other event conferring polymyxin B resistance sensitized the cells to deoxycholate.

### The Status of Position 1 of Lipid A Is Critical for CAMPs *versus* Deoxycholate Resistance

**FIGURE 7:**
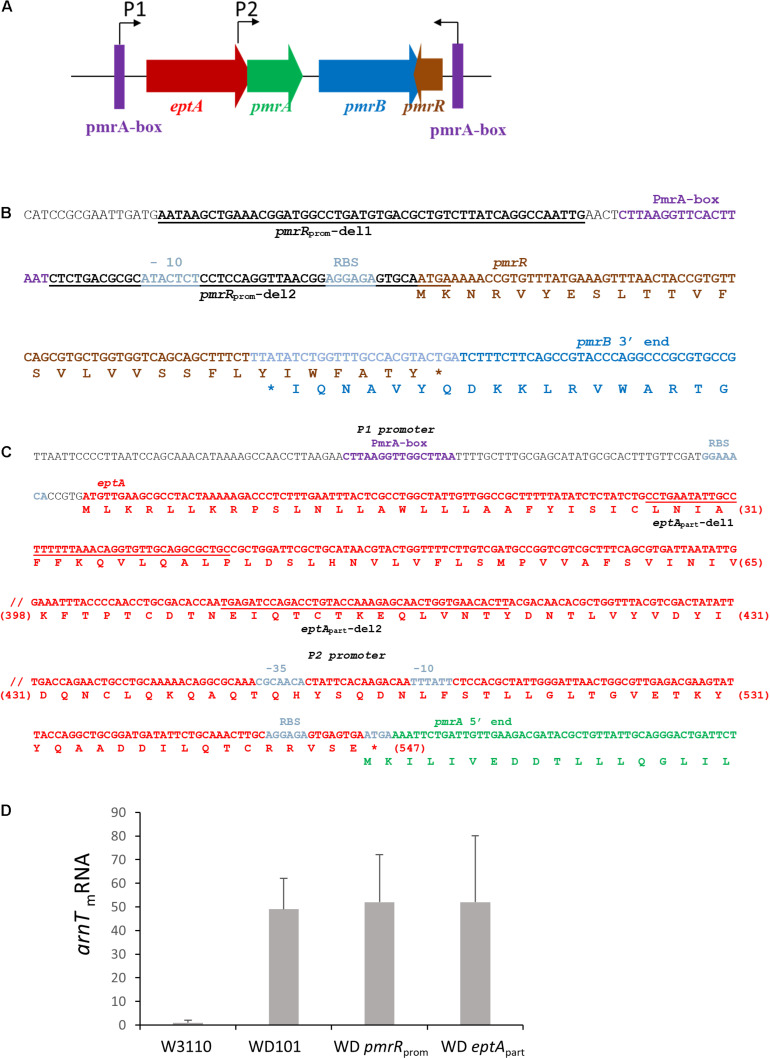
Construction of deletion mutants within the *eptA-pmrAB-pmrR* locus. **(A)** Schematic representation of the *eptA-pmrAB* operon and the divergently transcribed and overlapping *pmrR* gene. The expression of *eptA-pmrAB* operon is controlled by a PmrA-dependent promoter P1 (PmrA-box) and an internal promoter P2, while the expression of *pmrR* is controlled by a PmrA-dependent promoter (PmrA-box). The WD *pmrR*_*prom*_ and WD *eptA*_*part*_ deletion strains were generated by the Datsenko and Wanner method (Datsenko and Wanner, 2000). **(B)** DNA sequence of *pmrR* gene and the overlapping 3′-end sequence of *pmrB*. The regions of hybridization of primers *pmrR*_*prom*_-del1 and *pmrR*_*prom*_-del2 used for the deletion are underlined. The amino acid sequences are indicated below the DNA sequence. **(C)** DNA sequence of *eptA* gene and the overlapping 5′-end sequence of *pmrA*. The regions of hybridization of primers *eptA*_*part*_-del1 and *eptA*_*part*_-del2 used for the deletion are underlined. The amino acid sequences are indicated below the DNA sequence and the amino acid position within EptA sequence is indicated between brackets. **(D)** Q-PCR analysis of the transcriptional level of *arnT* gene in W3110, WD101, WD *eptA*_*part*_, and WD *pmrR*_*prom*_ strains. Total RNAs were extracted from bacterial cells grown in 2YT medium up to OD_600__*nm*_ = 0.5 and they were used for cDNA synthesis. The level of *arnT* mRNA was quantified by Q-PCR and normalized using housekeeping genes. Data correspond to the mean of three independent experiments.

**FIGURE 8:**
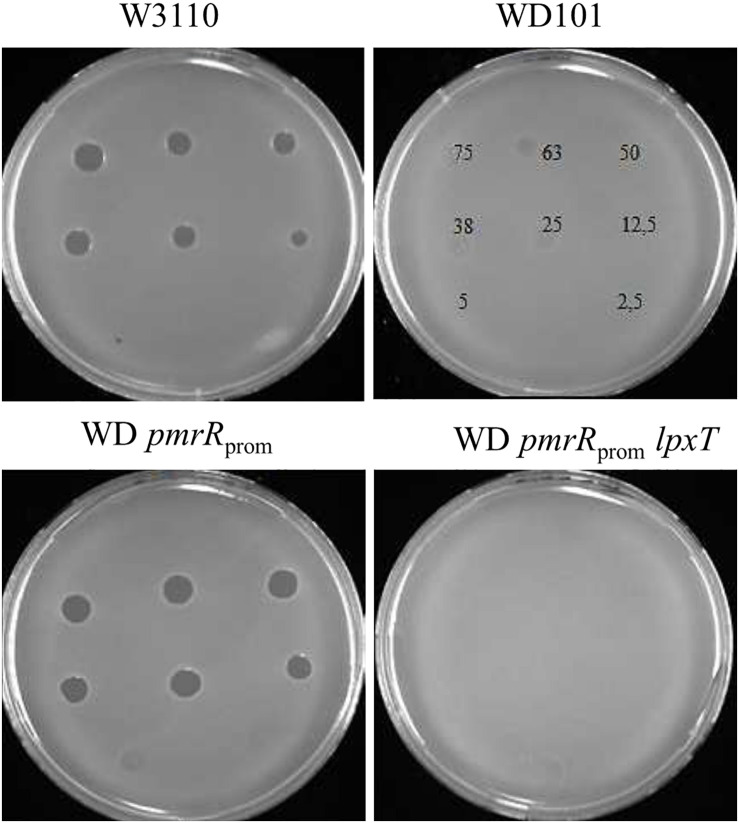
Phenotypic analysis of WD *pmrR*_*prom*_ strain and derivatives. 5-μl spots containing various amounts of polymyxin B (from 2.5 to 75 ng) were deposited on lawns of W3110, WD101, WD *pmrR*_*prom*_, and WD *pmrR*_*prom*_*lpxT* strains. Growth inhibition zones were observed after 16 h of incubation at 37°C. The data are representative of at least three independent experiments.

**FIGURE 9:**
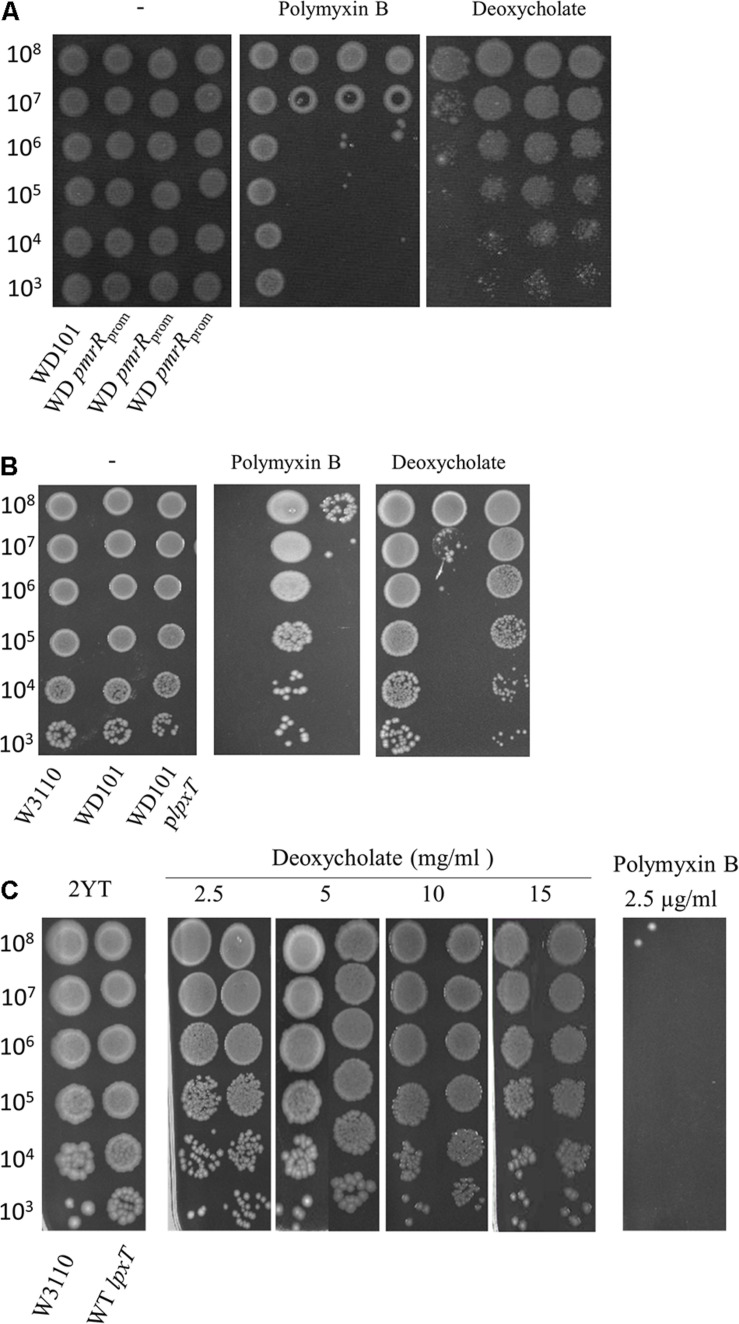
Effect of LpxT expression/activity on polymyxin B and deoxycholate susceptibility. **(A)** WD101 and three individual clones of WD *pmrR*_*prom*_ strain were grown overnight in 2YT medium and 5-μl spots of serial dilutions were deposited on 2YT plates supplemented or not with polymyxin B (2.5 μg/ml) and deoxycholate (2.5 mg/ml). **(B)** W3110, WD101, and WD101 carrying the plasmid p*lpxT* were grown overnight in 2YT medium and 5-μl spots of serial dilutions were deposited on 2YT plates supplemented or not with polymyxin B (2.5 μg/ml) and deoxycholate (2.5 mg/ml) **(C)**. W3110 and WT *lpxT* strains were grown overnight in 2YT medium and 5-μl spots of serial dilutions were deposited on 2YT plates supplemented or not with polymyxin B (2.5 μg/ml) and deoxycholate at various concentrations as indicated. The data are representative of at least three independent experiments.

Contrary to CAMPs that are positively charged, bile acids are negatively charged. Thus, deoxycholate susceptibility could arise from a decrease of LPS negative charge under PmrAB-inducing conditions, either due to the addition of positively charged groups and/or the decrease of lipid A 1-PP species. We then inactivated *pmrR* in WD101 strain to assess whether the lack of LpxT inhibition restored deoxycholate resistance. The *pmrR* gene is divergently transcribed from the *eptA-pmrAB* operon and the 3′ end of *pmrR* overlaps with *pmrB* (Figures 7A,B). In order to maintain the expression of *eptA-pmrAB* operon, we only deleted the PmrA-box from the *pmrR* promoter (Figure 7B). The WD *pmrR*_*prom*_ mutant developed polymyxin B susceptibility in contrast to its parental strain, and the further deletion of *lpxT* restored polymyxin B resistance (Figure 8). These findings were consistent with the expected lipid A modifications following the relief of LpxT from PmrR inhibition in WD101 strain (i.e., phosphorylation instead of addition of pEtN) according to Herrera et al. (2010). These results thus confirmed that the polymyxin B susceptibility displayed by WD *pmrR*_*prom*_ is due to the lack of PmrR and not to a side effect on the expression of *eptA-pmrAB*. The functionality of *eptA-pmrAB* was also tested by monitoring the expression of *arnT*, which is under the control of PmrA. As expected, the *arnT* transcript was 50-fold higher in WD101 and WD *pmrR*_*prom*_ strains as compared to WT (Figure 7D). Moreover, an ectopic copy of *pmrR* restored polymyxin B resistance in WD *pmrR*_*prom*_ strain (data not shown). Contrary to its parental strain, the WD *pmrR*_*prom*_ strain displayed deoxycholate resistance (Figure 9A). Moreover, *in trans* expression of *lpxT* in WD101 also restored deoxycholate resistance, while conferring polymyxin B susceptibility (Figure 9B). We tested whether LpxT was required for deoxycholate resistance of WT; however, W3110 *lpxT* strain did not show any susceptibility up to 15 mg/ml of deoxycholate (Figure 9C). In conclusion, when we restored LpxT activity in WD101, we abolished polymyxin B resistance and, conversely, we restored deoxycholate resistance. Therefore, the deoxycholate susceptibility of WD101 is due to either a lack of LpxT-modification, the presence of EptA-modification, since both modifications occur at the same position of lipid A in a competitive manner (Herrera et al., 2010; Kato et al., 2012; Figure 1A), or both events.

### The Inhibition of LpxT Accounts for Deoxycholate Susceptibility in PmrA Constitutive Strain

**FIGURE 10:**
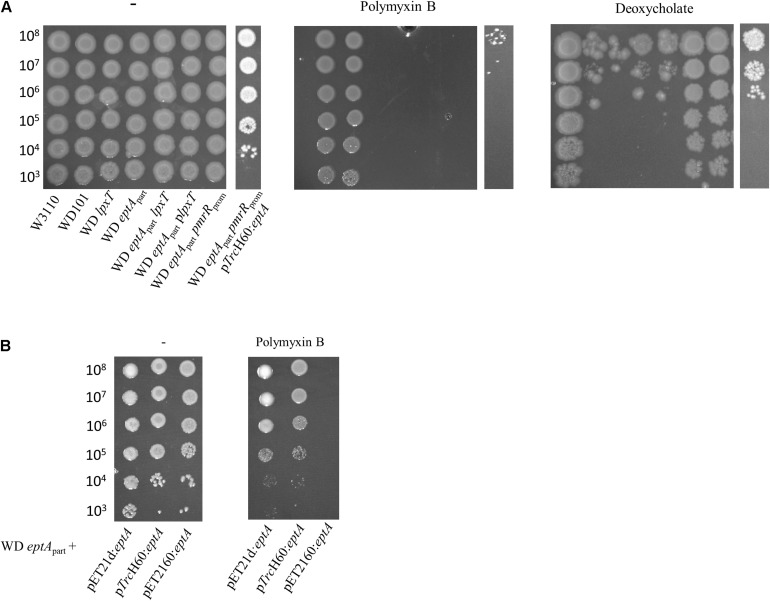
Phenotypic analysis of WD *eptA*_*part*_ strain and its derivatives. **(A)** WD *eptA*_*part*_ strain and its derivatives were grown overnight in 2YT medium and 5-μl spots of serial dilutions were deposited onto 2YT plates containing or not polymyxin B (2.5 μg/ml) and deoxycholate (2.5 mg/ml). **(B)** Functional complementation assays with plasmids carrying *eptA* gene under the control of its own promoter (pET21d:*eptA*), a *trc* promoter (p*Trc*H60:*eptA*) or a T7 promoter (pET2160:*eptA*). Cells were grown overnight overnight in 2YT medium and 5-μl spots of serial dilutions were deposited onto 2YT plates containing or not polymyxin B (2.5 μg/ml). The data are representative of at least three independent experiments.

To address this issue further, we inactivated *eptA* in WD101 strain. The expression of *eptA-pmrAB* operon relies on two promoters: a PmrA-dependent promoter located upstream of *eptA* and a constitutive promoter located at the 3′ end of *eptA*, which overlaps with the 5′ end of *pmrA* (Figures 7A,C; Soncini and Groisman, 1996; Lee et al., 2004). Therefore, to maintain *pmrAB* expression, we generated the WD *eptA*_*part*_ strain, which held 120-nt and 420-nt at the 5′ and 3′ ends of *eptA* open reading frame, respectively. The level of *anrT* transcript was similar in WD *eptA*_*part*_ and WD101 strains, i.e., 50-fold more than WT, attesting *pmrAB* functionality (Figure 7D). As was expected for a lack of pEtN decoration, which was previously demonstrated with a similar mutant (Herrera et al., 2010), WD *eptA*_*part*_ displayed polymyxin B susceptibility (Figure 10A). We tested functional complementation with plasmids carrying an ectopic copy of *eptA* under the control of its own promoter (pET21d:*eptA*), a *trc* promoter (p*Trc*H60:*eptA*) or a T7 promoter (pET2160:*eptA*). The plasmids pET21d:*eptA* and p*Trc*H60:*eptA* restored polymyxin B resistance in WD *eptA*_*part*_ strain (Figure 10B). Collectively, these data supported that WD *eptA*_*part*_ strain remained PmrA-constitutive, while EptA activity was lost.

Interestingly, WD *eptA*_*part*_ strain displayed similar deoxycholate susceptibility as WD101 (Figure 10A), supporting that the addition of pEtN *per se* does not confer deoxycholate susceptibility and, thus the lack of lipid A 1-PP, due to the inhibition of LpxT by PmrR, is directly responsible for bile acid susceptibility. We generated the WD *eptA*_*part*_ *pmrR*_*prom*_ strain, which exhibited deoxycholate resistance (Figure 10A). The resistance was also restored in WD *eptA*_*part*_ upon *in trans* expression of *lpxT* (Figure 10A). The plasmid p*Trc*H60:*eptA* restored deoxycholate susceptibility in WD *eptA*_*part*_ *pmrR*_*prom*_ likely due to the fact that overexpressed EptA outcompetes LpxT to modify lipid A at position 1. Meanwhile, it did not restore polymyxin B resistance (Figure 10A), suggesting that not enough pEtN decorations have been completed with respect to the CAMP. The WD *eptA*_*part*_ *lpxT* strain displayed sensitivity to both polymyxin B and deoxycholate as WD *eptA*_*part*_ (Figure 10A), demonstrating that according to polymyxin B, it is the lack of pEtN that conferred susceptibility and not the LpxT-dependent modification that may occur in the absence of EptA.

### LpxT Hinders Polymyxin B Resistance in WD *arnT* Strain

**FIGURE 11:**
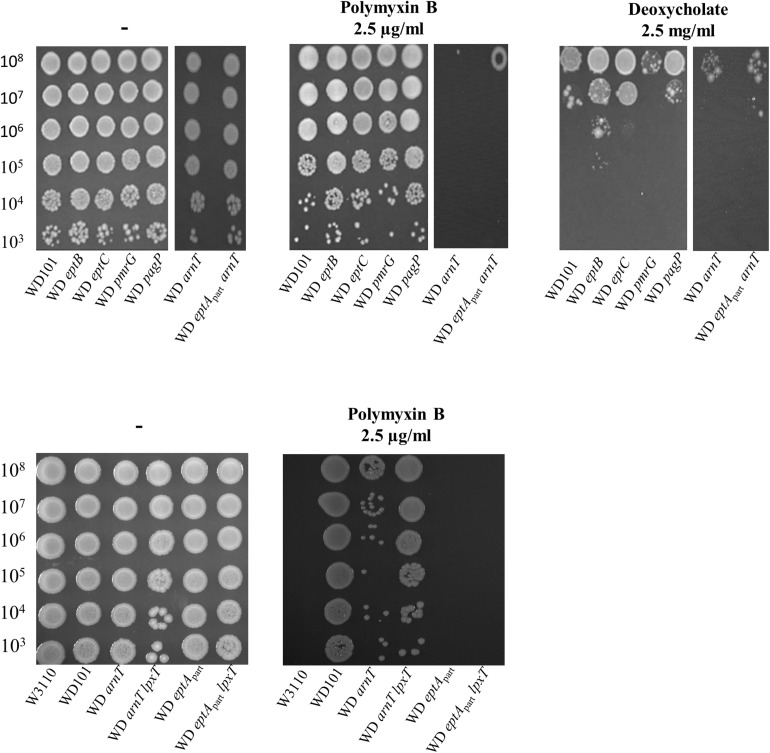
Phenotypic analysis of PmrA constitutive strain and derivatives. WD101 and its derivatives, deleted for various genes encoding different LPS-modifying enzymes, were grown overnight in 2YT medium and 5-μl spots of serial dilutions were deposited onto 2YT plates containing with or without polymyxin B (2.5 μg/ml) and deoxycholate (2.5 mg/ml). The data are representative of at least three independent experiments.

We further addressed the effect of other LPS modifications according to polymyxin B and deoxycholate by individually deleting *arnT*, *eptB*, *eptC*, *pmrG*, and *pagP* in WD101 strain. In accordance with Herrera et al. (2010), WD *arnT* mutant displayed increased polymyxin B susceptibility similar to WD *eptA*_*part*_ and WD *pmrR*_*prom*_ strains and all other mutants displayed polymyxin B resistance (Figure 11). These data indicated that the charges displayed at the level of lipid A play a determinant role in CAMPs resistance, while the charges displayed at the core level have no effect at least taken individually. All these mutants displayed deoxycholate susceptibility as WD101 and in contrast to WD *pmrR*_*prom*_ (Figure 11). Notably, WD *eptA*_*part*_ *arnT* also displayed deoxycholate susceptibility, supporting that only the lack of diphosphate group at position 1, due to the inhibition of LpxT by PmrR, is responsible for deoxycholate susceptibility. Unexpectedly, the inactivation of *lpxT* in WD *arnT* mutant restored polymyxin B resistance (Figure 11). This observation indicated that LpxT, whose enzymatic activity is inhibited by PmrR in WD101 background (Touzé et al., 2008), somehow hindered polymyxin B resistance in WD *arnT* strain.

### The Presence and Modality of O-Antigen Synthesis Have Not Effect on Deoxycholate Susceptibility

**FIGURE 12:**
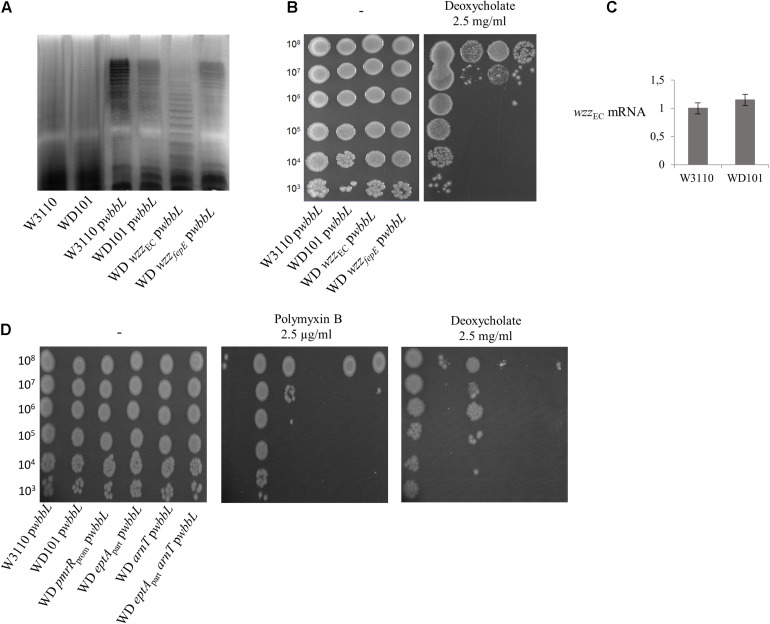
Restoration of O-antigen biosynthesis does not affect deoxycholate and polymyxin B susceptibility. **(A)** O-antigen biosynthesis was restored in W3110, WD101 and its derivatives upon expression of *wbbL* gene (p*wbbL* plasmid). Bacteria were grown overnight in 2YT medium and their LPS was extracted according to Crawford et al. (2012), resolved by 15% SDS-polyacrylamide gel electrophoresis and visualized by silver-staining. **(B,D)** Bacteria expressing *wbbL* gene were grown overnight in 2YT medium and 5-μl spots of serial dilutions were deposited onto 2YT plates containing or not polymyxin B (2.5 μg/ml) and deoxycholate (2.5 mg/ml). **(C)** Q-PCR analysis of the transcriptional level of *wzz*_*EC*_ and *wzz*_fepE_ genes in W3110 and WD101 strains. Total RNAs were extracted from bacterial cells grown in 2YT medium up to OD_600__*nm*_ = 0.5 and they were used for cDNA synthesis. The level of mRNA was quantified by Q-PCR and normalized using housekeeping genes. The data are representative of at least three independent experiments.

In *Salmonella*, the deoxycholate susceptibility of the PmrA-constitutive strain was found to rely on the expression of *wzz*_ST_, whose product controls the modality of O-antigen synthesis favoring the abundance of long chains (16 to 35 subunits) (May and Groisman, 2013; Islam and Lam, 2014). In contrast, very long O-antigen (>100 subunits) or the addition of pEtN and L-Ara4N had no effect on deoxycholate susceptibility (van Velkinburgh and Gunn, 1999; May and Groisman, 2013). The LPS of *E. coli* W3110 lacks the O-antigen chain due to a disruption of *wbbL* gene. We then examined whether restoring the O-antigen biosynthesis in *E. coli* and varying the modality of its synthesis could modulate deoxycholate susceptibility in a way comparable to *Salmonella*. SDS-PAGE analysis of LPS extracted from WT and WD101 carrying an ectopic copy of *wbbL* showed a restored synthesis of the O-antigen moiety (Figure 12A). As judged from the SDS-PAGE, the latter polymer mostly exhibited about 25 subunits and could therefore be qualified as long. The expression of *wbbL* and thus the abundance of these long O-antigens did not modify the phenotypes of WD101 and WT strains that remained susceptible and resistant to deoxycholate, respectively (Figure 12B). We assessed whether *wzz*_*EC*_ expression was upregulated in WD101 as compared to WT by monitoring *wzz*_*EC*_ transcript; however, w*zz*_*EC*_ was expressed at similar levels in both strains (Figure 12C). Thus, contrary to *Salmonella*, *wzz*_*EC*_ is not under the control of PmrA. This observation was correlated with the existence of similar O-antigens pattern (i.e., long O-antigens) in both WD101 and WT. Moreover, the inactivation of *wzz*_*EC*_ in WD101 led to the production of a majority of shorter O-antigens, further demonstrating the functionality of Wzz_*EC*_ (Figure 12A). The WD *wzz*_*EC*_ strain, which exhibited drastically less long O-antigen, remained deoxycholate susceptible (Figure 12B). In contrast to *Salmonella*, *wzz*_fepE_ was not upregulated upon PmrA activation as no *wzz*_fepE_ transcript was observed in WD101 or WT, which correlates with similar O-antigen patterns in both strains (Figure 12A). To investigate whether the presence of O-antigen changes the requirement for lipid A-1PP to resist deoxycholate in PmrA-constitutive background, the phenotype of a series of WD101 mutants carrying *wbbL* was examined. All mutants remained deoxycholate susceptible, except WD *pmrR*_*prom*_ (Figure 12D), demonstrating that in *E. coli*, the presence and the modality of the O-antigen synthesis have no major effect with respect to deoxycholate contrary to *Salmonella*.

### *lpxT* Expression Is Stimulated at Host Temperature

In *P. aeruginosa*, the *lpxT* gene transcript was identified in a screen for mRNA whose translation depends on temperature. The translation is blocked by a secondary structure that disassembles when temperature rises from 25 to 37°C (Delvillani et al., 2014). Although the 5’ UTR regions of *lpxT* from *P. aeruginosa* and *E. coli* do not present any similarity, we tested whether *lpxT* is also under the control of temperature in *E. coli*. We monitored the level of 3 × Flag-tagged LpxT in WT background after 1 h growth in 2YT-medium at 25, 30, 37, and 42°C. LpxT protein was present at a low level at 25°C and significantly increased with temperature rising: 2.9 and 3.6-fold increases were observed at 37 and 42°C, respectively, compared to 25°C (Figure 6B).

### LpxT Enhances Gut Colonization

**FIGURE 13:**
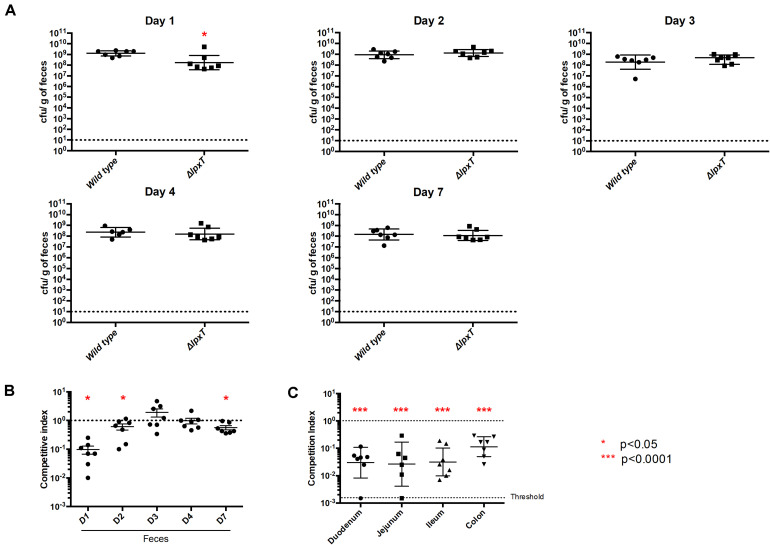
LpxT inactivation impairs colonization of mice. Groups of 7 OF1 mice were infected by gavage by MG1655 and MG *lpxT* (Cm^*R*^) strains (2 × 10^8^ bacteria per mouse) either separately **(A)** or as a mixture of both bacterial strains in equal proportions **(B,C)**. **(A)** Colonization rates were determined after 1, 2, 3, 4, and 7 days post-infection by enumeration of CFU per gram of gut. **(B)** Colonization rates were determined by the enumeration of CFU within the feces on LB-agar plates supplemented or not with chloramphenicol, after 1, 2, 3, 4, and 7 days post-infection. The results were expressed as the ratio of Cm^*R*^ bacteria out of the total recovered bacteria (competitive index). **(C)** Competitive index were determined by enumeration of bacteria (total and Cm^*R*^) in different regions of the gut after 1 day post-infection. Circles and squares represent individual mice while mean colonization levels are illustrated by horizontal bars. As indicated by red asterisks (*p < 0.05; ***p < 0.0001), the *lpxT* mutant showed a statistically significant defect in colonization when compared to the wild type strain in both separate and co-infection experiments. Data from two independent cohorts of mice were combined to increase significance and robustness of the analysis.

Such a temperature-dependent profile suggested a role of LpxT within the host. We then investigated a potential role in colonization of the mammalian gut. The *lpxT* deletion was introduced in the mouse-adapted *E. coli* WT strain MG1655 and the kinetics of colonization of WT and *lpxT* strains was followed in the feces from day 1 to day 7 post-oral challenge. WT was recovered in a significantly higher number as compared to *lpxT* strain (*p* < 0.05) from feces at day 1 and this gap was no longer visible at day 2 (Figure 13A). Of note, the maximal bacterial load in the feces was observed at day 1 for WT and this number decreased throughout the experiment (from 2 × 10^9^ cfu/g down to 1 × 10^8^ cfu/g). We performed competition experiments by inoculating WT and *lpxT* strains in an equal mixture. The WT was recovered from feces in much higher numbers at day 1 (about 25-fold in excess), also at days 2 and 4 but to a lesser extent (Figure 13B). We then monitored bacterial loads at different parts of the gut in the competitive assay after 1 day post-inoculation. Panel C shows that it was especially in the small intestine (duodenum, jejunum and ileum) that we observed at day 1 the biggest and equivalent disadvantage of the *lpxT* mutant (about 1.5–2 log; 50 to 100 times less), while only a 1 log of difference (10 times less) (*p* < 0.0001) was observed in the colon (Figure 13C). All this suggested that LpxT rather brought an advantage in the initial stages of colonization where *E. coli* must pass through areas with high bile acid concentrations.

## Discussion

In their natural habitat, bacteria have to cope with various noxious compounds with sometimes opposite chemical properties such as CAMPs and bile acids. CAMPs are abundant in nature; some are produced by host innate immune systems to prevent invading pathogens. Polymyxins are cyclic CAMPs that are used as last resort antibiotics due to their toxicity in mammals (Falagas and Michalopoulos, 2006; Biswas et al., 2012). Nevertheless, the growing threat of multi-resistant bacteria has renewed therapeutic interest in these molecules. Clinical isolates that are resistant to polymyxins display either mutations in PhoPQ or PmrAB TCS promoting high level of lipid A decorations or mobile genetic elements carrying MCR genes encoding EptA homologs (Olaitan et al., 2014; Zhang et al., 2019). Nevertheless, we showed here that WT *E. coli* K-12 resist to 2.5 μg/ml of polymyxin B provided that both PhoPQ and PmrAB TCS are stimulated by environmental cues. The presence of high concentrations of iron combined with mildly acidic pH was required to elicit this phenotype. Irrespective of the conditions, the activation of PhoPQ was required, meaning that specific gene(s) under the control by PhoPQ needed. WD101 strain, which is PmrA-constitutive, still required the presence of a stimulus recognized by PhoP to display full polymyxin B resistance. PhoP upregulates PagP, which adds an acyl chain to lipid A; however, the disruption of *pagP* in WD101 did not compromise polymyxin B resistance. We showed that the PmrD connector is functional in conferring the proper amplification of PmrAB activity, but PmrD became unessential when both PhoPQ and PmrAB were directly stimulated in conditioning and challenging milieus. This study highlighted the complexity and the extent of the signals that are required to elicit CAMPs resistance and that TCS undergo a fine-tuned level of activation. The degree of stimulation must then be adapted to the constraint encountered by the bacterial cells. This is critical when one particular modification is beneficial to one constraint (e.g., the presence of CAMPs) but detrimental to another (e.g., the presence of bile acids). This conflict may then occur during gut colonization in mammalian hosts where PmrAB-inducing conditions are encountered together with bile acids (Merighi et al., 2005).

Among LPS decorating enzymes, we found that both EptA and ArnT were critical to confer polymyxin B resistance, while the enzymes responsible for the core oligosaccharide modifications, the addition of palmitate to lipid A or the presence and the modality of O-antigen synthesis did not have any effect. Unexpectedly, we observed that the presence of LpxT in WD101 hindered polymyxin B resistance upon ArnT inactivation since WD *arnT lpxT* strain appeared polymyxin B resistant, while WD *arnT* was susceptible. This suggested that the lipid A decoration with L-Ara4N *per se* was not essential for polymyxin B resistance. Notably, in WD101 background, the LpxT-dependent modification was no more accomplished due to the production of PmrR (Touzé et al., 2008). Herrera et al. (2010) earlier reported that the WD *arnT* strain produced lipid A species modified with pEtN at both 1 and 4′ positions. The polymyxin B susceptibility of WD *arnT* strain indicated that this alternate pEtN modification at the 4′ position did not compensate for the loss of L-Ara4N. These authors also reported that, in PmrA-inducing conditions, the *E. coli* MST01 *lpxT* strain displayed an important gain of pEtN modification at both 1 and 4′ positions, with a concomitant loss of L-Ara4N as compared to the WT (Herrera et al., 2010). Together, this suggested that upon *lpxT* and *arnT* disruption in WD101, pEtN modification occurred at both 1 and 4′ positions, which could thus overcome the lack of L-Ara4N and confer polymyxin B resistance. These observations suggested that LpxT, whose enzymatic activity is blocked by PmrR, likely reduced the activity of EptA in targeting the 4′ position. This phenomenon likely implies an interaction of LpxT/PmrR complex with EptA or other lipid A-decorating enzymes.

The constitutive activation of PmrA in *E. coli* DW137 strain was previously found to confer deoxycholate susceptibility (Froelich et al., 2006). Here, we showed that the stimulation of PmrAB indeed compromised survival of *E. coli* exposed to bile acids. We demonstrated that the inhibition of LpxT by PmrR accounted for the loss of resistance to deoxycholate in the PmrA-constitutive background. This is the first time that a function was assigned to LpxT in *E. coli*. Moreover, we highlighted the necessity for *E. coli* to fine-tune the status of position 1 of their lipid A to resist major antibacterial agents present in the intestine of mammals. The presence of lipid A-1PP increases the net negative charge at the cell surface, which likely decreases the affinity of the membrane to the negatively charged bile acids. The presence of a certain content of lipid A-1PP must then be critical to cope with the presence of bile. Of note, the lack of both ArnT and pEtN decorations in a PmrA-constitutive background did not restore deoxycholate resistance, contrary to the restoration or relief of LpxT from PmrR inhibition.

We further showed that the inactivation of *lpxT* impaired the kinetics of gut colonization of *E. coli* in mouse. In the early stages of colonization, *E. coli* must pass and establish itself throughout the intestinal tract. The default of *lpxT* mutant may arise from the susceptibility of this strain to bile acids that are more abundant in the small intestine. It is known that bile acids modulate the commensal flora particularly in the small intestine. But once a few mutants get to the colon, they eventually become permanently established because bile acid concentrations are much lower there. Hence a lack of (big) difference between WT and mutant in competition in the feces beyond day 1 post-oral challenge. CFU/feces reflects the colonization status of the colon and cecum rather than the small intestine. This was also reflected by the mono-colonization that showed an effect only at day 1. Our results showed that the biggest and equivalent disadvantage of the *lpxT* mutant as compared to the WT occurred in the small intestine (duodenum, jejunum, and ileum), while in the colon we have only a small difference at day 1. All this suggested that LpxT brings an advantage in the initial stages of colonization where *E. coli* must pass through areas with high bile acid concentrations. It should be highlighted that our mouse model favors the implantation of *E. coli* since we eliminate the aerobic flora with the streptomycin treatment. The bacterial loads achieved by *E. coli* are higher than expected during natural infection or colonization of the intestinal tract of permissive animals. Hence, the difference we observe could be amplified during natural colonization due to a stronger bottle neck. We also showed that the expression of *lpxT* was significantly increased at the host temperature, supporting a role in colonization. The temperature-dependent expression of *lpxT* from *P. aeruginosa* was previously reported. Interestingly, the 5′ UTR of *lpxT* mRNA from *E. coli* was predicted to adopt a FourU-like structure that is different from the putative structure of the 5′UTR of *lpxT* from *P. aeruginosa* (Delvillani et al., 2014). The fact that the same regulation pattern was achieved by various means in distant bacterial species strengthens the physiological significance of this regulatory control.

The *Salmonella* PmrA-constitutive strain also displayed deoxycholate susceptibility, but in this bacterium, the upregulation of *wzz*_ST_, which controls the modality of the O-antigen synthesis, was responsible of this phenotype (Ramos-Morales et al., 2003; May and Groisman, 2013). It was then relevant to question whether restoring O-antigen synthesis and varying its length in *E. coli* could modulate deoxycholate susceptibility. However, whichever the modality of the O-antigen synthesis, in particular when *wzz*_*EC*_ was inactivated (i.e., when mostly short O-antigens were produced), WD101 strain remained deoxycholate susceptible unless LpxT activity was restored through *pmrR* disruption. Interestingly, the *wzz*_ST_ gene product from *Salmonella* was also shown to control the proper balance between L-Ara4N and pEtN modifications on lipid A. A *wzz*_ST_ mutant displayed pEtN modification at both 1 and 4′ positions in PmrA-inducing conditions suggesting that the Wzz_ST_ protein exerts a negative control over EptA allowing the modification by ArnT at the 4′ position (Farizano et al., 2012). Thus, the deoxycholate susceptibility observed in *Salmonella* under PmrA-inducing conditions might not originate from the modality of the O-antigen synthesis *per se* but rather from an alternate lipid A decoration pattern. This control of the lipid A modifying enzymes by Wzz homolog did not seem to occur in *E. coli*. Whether LpxT is also involved in deoxycholate resistance in *Salmonella* under PmrA-inducing conditions should now be addressed at the light of this study.

## Materials and Methods

### Bacterial Strains, Plasmids, Media

Bacterial strains and plasmids are listed in Table 1. Primers are listed in the Supplementary Material. Bacteria were grown at 37°C in 2YT broth or N-min minimal medium (pH 7.5 or 5.8) containing 0.1% casamino acids and 38 mM glycerol (Chamnongpol et al., 2002), and the indicated concentrations of MgCl_2_ and FeSO_4_. When required, the medium was supplemented with ampicillin, kanamycin or chloramphenicol at 100, 50, and 25 μg/ml, respectively. The different enzymes used for molecular biology techniques were from New England Biolabs, and DNA purification kits were from Macherey-Nagel. Primer synthesis and DNA sequencing were performed by Eurofins-MWG. All other materials were reagent grade and obtained from commercial sources.

**TABLE 1:** Bacterial strains and plasmids.

Strains	Genotype or description	References
W3110	K-12 F^–^ λ^–^ *rph-1* INV(*rrnD*, *rrnE*)	*E. coli* Genetic Stock Center (Yale)
MG1655	K-12 F^–^ λ^–^ *ilvG*^–^ *rfb-50 rph-1*	*E. coli* Genetic Stock center (Yale)
MG *lpxT*	MG1655 Δ*lpxT*:Cm^*R*^	This study
WD101	W3110 PmrA constitutive (PmrA^*c*^)	Trent et al., 2001
DMEG3	BW25113 Δ*lpxT*:Cm^*R*^	El Ghachi et al., 2005
JW2251	BW25113 Δ*arnT*:Kan^*R*^	Baba et al., 2006
JW5660	BW25113 Δ*eptB*:Kan^*R*^	Baba et al., 2006
JW3927	BW25113 Δ*eptC*:Kan^*R*^	Baba et al., 2006
JW2246	BW25113 Δ*pmrG*:Kan^*R*^	Baba et al., 2006
JW0617	BW25113 Δ*pagP*:Kan^*R*^	Baba et al., 2006
JW5836	BW25113 Δ*wzz*_*EC*_:Kan^*R*^	Baba et al., 2006
JW0579	BW25113 Δ*wzz*_fepE_:Kan^*R*^	Baba et al., 2006
JW1116	BW25113 Δ*phoP*:Kan^*R*^	Baba et al., 2006
JW2254	BW25113 Δ*pmrD*:Kan^*R*^	Baba et al., 2006
JW4074	BW25113 Δ*pmrA*:Kan^*R*^	Baba et al., 2006
BW *lpxT*-Flag	BW25113 *lpxT*-Flag:Kan^*R*^	This study
WT *lpxT*-Flag	W3110 *lpxT*-Flag:Kan^*R*^	This study
WT *phoP lpxT-*Flag	W3110 Δ*phoP lpxT*-Flag:Kan^*R*^	This study
WD *lpxT-*Flag	WD *lpxT*-Flag:Kan^*R*^	This study
WT *phoP*	W3110 Δ*phoP*	This study
WT *pmrD*	W3110 Δ*pmrD*	This study
WT *lpxT*	W3110 Δ*lpxT*	This study
WD *eptA*_*part*_	WD101 Δ*eptA*_*part*_	This study
WD *eptA*_*part*_ *lpxT*	WD101 Δ*eptA*_*part*_Δ*lpxT*	This study
WD *pmrR*_*prom*_	WD101 Δ*pmrR*_*prom*_	This study
WD *eptA*_*part*_ *pmrR*_*prom*_	WD101 Δ*eptA*_*part*_Δ*pmrR*_*prom*_	This study
WD *arnT*	WD101 Δ*arnT*	This study
WD *eptA*_*Part*_ *arnT*	WD101 Δ*eptA*_*part*_Δ*arnT*	This study
WD *arnT lpxT*	WD101 Δ*arnT*Δ*lpxT*	This study
WD *eptB*	WD101 Δ*eptB*	This study
WD *eptC*	WD101 Δ*eptC*	This study
WD *pmrG*	WD101 Δ*pmrG*	This study
WD *pagP*	WD101 Δ*pagP*	This study
WD *wzz*_fepE_	WD101 Δ*wzz*_fepE_	This study
WD *wzz*_*EC*_	WD101 Δ*wzz*_*EC*_	This study
Plasmids		
pCP20	Resistance cassette removal by Flp recombinase expression, Amp^*R*^, Cam^*R*^	Datsenko and Wanner, 2000
pKD3	PCR amplification of Cam^*R*^ cassette for gene deletion, Cam^*R*^, Amp^*R*^	Datsenko and Wanner, 2000
pKD46	Resistance cassette insertion by lambda red recombinase expression, Amp^*R*^	Datsenko and Wanner, 2000
p*Trc*His60	p*Trc*99A derivative, Amp^*R*^	Pompeo et al., 1998
p*Trc*His30	p*Trc*99A derivative, Amp^*R*^	Pompeo et al., 1998
pET21d	Expression vector, Amp^*R*^	Novagen
pET2160	pET21d derivative, Kan^*R*^	Barreteau et al., 2009
pSUB11	R6K g ori 3 × Flag FRT Neo^*R*^/Kan^*R*^ FRT Amp^*R*^	Uzzau et al., 2001
p*lpxT*	p*Trc*His30 derivative for expression of *lpxT* generated by using *lpxT-Xba*I and *lpxT-Hin*dIII primers	This study
pET21d:*eptA*	pET21d derivative for expression of *eptA* generated by using *eptA-Hin*dIII and *eptA-Nco*I-3′ primers	This study
pET2160:*eptA*	pET2160 for expression of *eptA* generated by using *eptA-Nco*I-5′ and *eptA-Bgl*II primers	This study
p*Trc*H60:*eptA*	p*Trc*His60 derivative for expression of *eptA* generated by using *eptA-Nco*I-5′ and *eptA-Bgl*II primers	This study
p*wbbL*	pET21d derivative for expression of *wbbL*, Amp^*R*^	This study

### Strains Construction

WD *eptA*_*part*_, WD *pmrR*_*prom*_ and BW *lpxT*-Flag were generated by using the Datsenko and Wanner method (Datsenko and Wanner, 2000). For WD *eptA*_*part*_ and WD *pmrR*_*prom*_, the primers were designed to amplify the Cm^*R*^ resistance cassette from the template plasmid pKD3, flanked by 50 bp from the targeted chromosomal region for homologous recombination (Supplementary Table 1 and Figures 7B,C). To generate the BW *lpxT*-Flag strain, the primers (Supplementary Table 1) were designed to amplify the Kan^*R*^ resistance cassette and the 3 × Flag tag-coding sequence from the template plasmid pSUB11 (Uzzau et al., 2001), flanked by 50 bp from the targeted chromosomal region. The PCR products were transformed in the WD101 or BW25113 recipient strains harboring the pKD46 plasmid for λ red recombinase expression. After selection of the mutated strains under the appropriate selective pressure, the thermosensitive pKD46 plasmid was cured. For WD *eptA*_*part*_ and WD *pmrR*_*prom*_, the antibiotic resistance cassette was finally removed from the chromosome by using the pCP20 plasmid for expression of the FLP recombinase, yielding an 85-nt FRT scar in place of the deleted region (Datsenko and Wanner, 2000).

WT *lpxT*, WT *pmrD*, WT *phoP*, WD *arnT*, WD *eptB*, WD *eptC*, WD *pmrG*, WD *pagP*, WD *wzz*_*EC*_, WD *wzz*_fepE_, WD Δ*pmrR*_*prom*_ *lpxT*, WD *eptA*_*part*_ *lpxT*, WD *arnT lpxT*, WT *lpxT-*Flag, WT *phoP lpxT-*flag, and WT *pmrA lpxT-*Flag were generated by P1-mediated transduction by using the following strains as donors: DMEG3 (El Ghachi et al., 2005), BW25113-based Keio collection (Baba et al., 2006), or BW *lpxT*-Flag. The antibiotic resistance cassette was always removed by FLP recombination except for the *lpxT*-Flag strains. The MG *lpxT* strain was generated by P1 transduction using DMEG3 strain as donor and MG1655 strain as recipient strain. All the mutant strains were systematically controlled by PCR using appropriate primers (Supplementary Table 2).

### Plasmids Construction

The plasmids used for the expression of an ectopic copy of *lpxT* or *eptA* were generated by PCR amplification of the corresponding ORFs with primers listed in Supplementary Table 3, followed by their insertion in the appropriate expression vectors (Table 1). The primers and vectors which were used are specified in Table 1. For the construction of the plasmid for *wbbL* expression, the gene was amplified by overlap extension PCR in two steps. The two parts of the gene, flanking the inserted IS5 element, were PCR amplified from BW25113 chromosomal DNA with primers *wbbL-Nco*I and *wbbL*-mid1 (5′-end of the gene) and *wbbL-Hin*dIII and *wbbL*-mid2 (3′-end of the gene). The resulting PCR products were then used as templates in a second PCR for the amplification of the reconstituted gene, which was then inserted in the pET21d vector. All plasmids were checked by sequencing.

### Assay for Polymyxin B and Deoxycholate Susceptibility

The bacteria were grown overnight at 37°C in 2YT medium or N-min medium at pH 7.5 or 5.8, in the presence of MgCl_2_ (10 μM or 1 mM). When additional conditioning was performed as specified, the overnight culture was washed twice in fresh N-min medium pH 5.8 or 7.5 and the cells were diluted 1:50 in N-min at pH 7.5 or 5.8, 10 μM or 1 mM MgCl_2_ and 300 μM FeSO_4_ and subsequently incubated at 37°C under agitation for 4 h. The culture (overnight culture or conditioned cells) was used to prepare cellular suspensions at 10^3^ to 10^8^ CFU/ml in sterile water. The number of CFU of the culture was determined according to the OD_600__*nm*_ (1 unit corresponding to 3 × 10^8^ CFU/ml). 5-μl aliquots of these serial dilutions were deposited on 2YT or N-min agar plates containing or not polymyxin B and deoxycholate at the indicated concentrations, which were incubated at 37°C for 24 to 48 h.

Alternatively, the bacteria were grown overnight at 37°C in 2YT medium and a suspension at 10^8^ CFU/ml was prepared in 5 ml of sterile water. A 2YT agar plate was flooded with this suspension for 1 min to allow the bacteria to sediment before removing the excess of water. 5-μl drops of polymyxin B solutions at various concentrations were added at the surface of the plates, which were then incubated at 37°C for 16 h.

### Assay for Cell Survival to Deoxycholate Exposure

The bacteria were grown at 37°C overnight in 2YT medium or N-min medium at pH 5.8, 10 μM MgCl_2_. The respective cultures were diluted 1:50 in 2YT medium or in N-min at pH 5.8, 10 μM MgCl_2_ and 300 μM FeSO_4_ and subsequently incubated at 37°C with agitation for 4 h. Two suspensions at 10^5^ CFU/ml were prepared in PBS buffer to be challenged or not with deoxycholate at 10 mg/ml final concentration at 37°C with agitation for 1 h. The suspensions were diluted in PBS buffer and spread on 2YT agar plates for the enumeration of survivals, which was done after overnight incubation of the plates at 37°C.

### Analysis of LPS

The LPS were prepared according to the protocol already described (Crawford et al., 2012). They were analyzed by 15% SDS-polyacrylamide gel electrophoresis and visualized by silver staining as described previously (Tsai and Frasch, 1982).

### Quantitative RT-PCR Analysis

Total RNA were extracted from bacteria grown to the middle of exponential phase (OD_600__*nm*_ = 0.5) using RNeasy Protect bacteria Mini Kit system (Qiagen) according to the manufacturer’s instructions. cDNA synthesis was performed from 1 μg of total RNA with random hexanucleotides as primers using the Superscript IV First Strand Synthesis system for RT-PCR (Invitrogen). The quantitative PCR reactions were then carried out with the appropriate primers (Supplementary Table 4) using DyNAmo ColorFlash SYBR Green qPCR kit (Thermo Scientific) and they were run in a StepOnePlus Real-Time PCR system (Applied Biosystems). The data were analyzed with StepOne software v2.3 using ΔΔCt method and normalized using the housekeeping genes *rrsA*, *gyrA* and *ffh* as reference genes.

### Expression Analysis of 3 × Flag-Tagged LpxT

Bacterial strains expressing the 3 × Flag-tagged LpxT (WT *lpxT*-Flag, WT *pmrA lpxT*-Flag, and WT *phoP lpxT*-Flag) were grown overnight in 2YT at 37°C, diluted 100 fold in fresh 2YT medium and then incubated at 37°C until the OD_600__*nm*_ reached 0.7. To test the expression of Flag-tagged LpxT at different temperatures, the WT *lpxT*-Flag strain was grown overnight in 2YT at 25°C and diluted in four flasks containing fresh 2YT medium at an initial OD_600__*nm*_ = 0.2, which were then incubated at 25, 30, 37, or 42°C for 1 h under agitation. 1 ml of each culture was then used for total protein extraction. The cells were centrifuged and the pellet was resuspended in SDS buffer (50 mM Tris–HCl pH 6.8, 100 mM β-mercaptoethanol, 2% SDS, 0.1% bromophenol blue and 10% glycerol) and boiled for 15 min. Similar amounts of proteins, according to the OD_600__*nm*_ of the culture, were analyzed by 15% SDS polyacrylamide gel electrophoresis and transferred to a polyvinylidene difluoride membrane. The membrane was incubated first with monoclonal antibody Anti-Flag M2 (Sigma) and then with antibody Anti-IgG from mouse (Fc Specific) coupled to a peroxidase (Sigma). The blots were developed with ECL system (Bio-Rad) according to the manufacturer’s protocol. The images were collected with ImageQuant^TM^ LAS 500 (GE Healthcare-Life Sciences) and the proteins were quantified by Image Studio Lite by measuring the density of the corresponding bands.

### Colonization

OF1 female mice purchased from Charles River Laboratories and aged 5 weeks were given bottles of water with 5 g/L of streptomycin (treatment started 2 days prior to gavage) and infected by gavage with feeding needles with MG1655 strains (2 × 10^8^ bacteria per mouse) resistant to streptomycin. For the competitive assay, mice were infected by MG1655 and MG *lpxT* strains in equal proportions (2 × 10^8^ total bacteria per mouse). Colonization rates were determined by enumeration of CFU per gram of feces. The samples were diluted and spread onto 2YT agar plates with streptomycin (5 μg/ml) supplemented with 20 μg/ml of chloramphenicol to enumerate MG *lpxT* cells and/or without chloramphenicol to enumerate total *E. coli* cells (i.e., WT and *lpxT* mutant). Colonization rates were also determined by enumeration of CFU per gram of different parts of the gastrointestinal tract. Mice were euthanized with CO_2_, parts of the gut were ground and homogenized in peptone broth and MG1655 and MG *lpxT* cells were enumerated. The results of two independent colonization experiments (seven mice by cage) were pooled and a one tailed Mann-Whitney test was used to determine statistical significance of observed differences (GraphPad Prism v5.0 GraphPad Software, CA).

## Data Availability Statement

The original contributions presented in the study are included in the article/Supplementary Material, further inquiries can be directed to the corresponding author.

## Ethics Statement

The animal study was reviewed and approved by the animal experiments were done according to European (Directive 2010/63 EU) and French regulation (Décret 2013-118) under the authorized protocol CETEA 2014-072 reviewed by the Institut Pasteur Ethical Committee (registered as number 89 with the French Ministry of Research). The experimental protocol was also approved by the French Ministry of Research under the number APAFIS#11694-2017100510327765 v2.

## Author Contributions

XT, GM, and TT conceived and designed the study. XT, GM, EG, RA, and SH performed the experiments. XT, GM, TT, DM-L, and IB analyzed the results. TT wrote the manuscript. All authors reviewed and approved the final manuscript.

## Conflict of Interest

The authors declare that the research was conducted in the absence of any commercial or financial relationships that could be construed as a potential conflict of interest.

## Abbreviations

C_55_-P: undecaprenyl phosphate
C_55_-PP: undecaprenyl pyrophosphate
CAMPs: cationic antimicrobial peptides
L-Ara 4N: 4-amino-4-deoxy-L-arabinose
lipid A 1-PP: lipid A 1-diphosphate
LPS: lipopolysaccharide
pEtN: phosphoethanolamine
TCS: two component regulatory system.
