# Supplementary material for: LpxT-Dependent Phosphorylation of Lipid A in Escherichia coli Increases Resistance to Deoxycholate and Enhances Gut Colonization
Source: Front Microbiol. 2021 May 4;12:676596. doi: 10.3389/fmicb.2021.676596 (PMC8129183; doi:10.3389/fmicb.2021.676596)
Supplement: Supplementary file 1 [file Table_1.DOCX]

**Supplemental Material**

**LpxT-dependent phosphorylation of Lipid A in *Escherichia coli* increases resistance to deoxycholate and enhances gut colonization.**

**Xudong Tian^1^**^†^**, Guillaume Manat^1^**^†^**, Elise Gasiorowski^2,3^, Rodolphe Auger^1^, Samia Hicham^2^, Dominique Mengin-Lecreulx^1^ and Ivo Gomperts Boneca^2^, Thierry Touzé^1*^**

^1^Université Paris-Saclay, CEA, CNRS, Institute for Integrative Biology of the Cell (I2BC), 91198, Gif-sur-Yvette, France

^2^Institut Pasteur, Unité biologie et génétique de la paroi bactérienne. 28, rue du Docteur Roux, 75015 Paris, France; CNRS, UMR 2001 Microbiologie Integrative et Moléculaire, 75015 Paris, France; INSERM, Groupe Avenir, 75015 Paris, France.

^3^ Université de Paris, Sorbonne Paris Cité, Paris, France

^†^These authors have contributed equally to this work and share first authorship.

Keywords: lipopolysaccharides, lipid A, two-component system, antibiotic resistance, polymyxin B, bile acid

Running title: LpxT increases bile acid resistance

**^*^**Address correspondence to: Thierry Touzé, Université Paris-Saclay, CEA, CNRS, Institute for Integrative Biology of the Cell (I2BC), 91198, Gif-sur-Yvette, France

E-Mail: [thierry.touze@i2bc.paris-saclay.fr](mailto:thierry.touze@i2bc.paris-saclay.fr)

**Table S1.** Primers used for construction of deletion mutants via homologous recombination.

| Primers | Sequence (5'-3') |
| --- | --- |
| *eptA*_part_-del1 | CCTGAATATTGCCTTTTTTAAACAGGTGTTGCAGGCGC  TGCGTGTAGGCTGGAGCTGCTTC |
| *eptA*_part_-del2 | AAGTGTTCACCAGTTGCTCTTTGGTACAGGTCTGGATC  TCACATATGAATATCCTCCTTAG |
| *pmrR*_prom_-del1 | AATAAGCTGAAACGGATGGCCTGATGTGACGCTGTCTT  ATCAGGCCAATTGTGTAGGCTGGAGCTGCTTC |
| *pmrR*_prom_-del2 | TCATTGCACTCTCCTCCGTTAACCTGGAGGAGAGTATGC  GCGTCAGAGCATATGAATATCCTCCTTAG |
| *lpxT*-3×Flag-for | CCTTTTTTGACAAATCACTACCAGGAAAAAACAAACATT  TCCAAAACAAAGACTACAAAGACCATGACGG |
| *lpxT*-3×Flag-rev | ATCAGGATTATCCTCACTATAAAAATAACCCTGATGATGTTAATTACTGTCATATGAATATCCTCCTTAG |

**Table S2.** Primes used to control the genotype of deletion strains.

| *pmrR*-ctrl1 | AGCGTTTGTACGTATGGACA |
| --- | --- |
| *pmrR*-ctrl2 | CAGCAACATCCGCGAATTGA |
| *phoP*-ctrl1 | TAAACCTCGTATCAGTGCCG |
| *phoP*-ctrl2 | AGCGAAAGCACCAGTACTAC |
| *pmrD*-ctrl1 | TTCGCTGAAAGCACTACTGG |
| *pmrD*-ctrl2 | GCCATTCCATTGCCGTTGAT |
| *eptA*-ctrl1 | CTTTGCGAGCATATGCGCAC |
| *eptA*-ctrl2 | ACGCGTAGCCTTCGGTTTGC |
| *arnT*-ctrl1 | CGCTCGGACAAGTTGTTCGC |
| *arnT*-ctrl2 | ATCGCCACAAAGCAGGTTGC |
| *eptB*-ctrl1 | GGCTTCTATGTGACCTTCTA |
| *eptB*-ctrl2 | TCATCCTCATGAGCTGCAGA |
| *eptC*-ctrl1 | CTTCCTCTTCTGCAAACCCT |
| *eptC*-ctrl2 | AACGCCTTATCCAGCCTACC |
| *pagP*-ctrl1 | GACTATTCCCATCGCAGAAA |
| *pagP*-ctrl2 | CACCAATTGTGGTACGCTTT |
| *pmrG*-ctrl1 | TTGCCGACATCGTAGGATTT |
| *pmrG*-ctrl2 | AACCTGAAGATCTGGTGCAT |
| *wzz*_EC_-ctrl1 | AACGGCATCAGTGAGTTGCG |
| *wzz*_EC_-ctrl2 | ATCCTATAGCATTCACGAGG |
| *wzz*_fepE_-ctrl1 | ATGTTGCATTCAGTGGAAGG |
| *wzz*_fepE_-ctrl2 | GTCTGCGCTGCATGATCATT |

**Table S3.** Primers used for construction of plasmids^a^.

| *eptA*-NcoI-5’ | CCCCCCCATGGTGTTGAAGCGCCTACTAAAA |
| --- | --- |
| *eptA*-NcoI-3’ | CCCCCCCATGGTTTCATTCACTCACTCTCCT |
| *eptA*-BglII | CCCCCAGATCTTTCACTCACTCTCCTGC |
| *eptA*-HindIII | CCCCCAAGCTTGGTCATTTGTCACCCTGATG |
| *wbbL*-NcoI | CCCCCCATGGTATATATAATAATCGTTTCCC |
| *wbbL*-mid1 | AGGAATTTTTGTTTTATTAATCCCTAACATAAATGACACA |
| *wbbL*-HindIII | CCCCCAAGCTTTTACGGGTGAAAAACTGATGAAATTC |
| *wbb*L-mid2 | TGTGTCATTTATGTTAGGGATTAATAAAACAAAAATTCCT |
| *lpxT*-XbaI | CGCGTCATGATGCCTGCGTTTTTCAGTAAGATAATT |
| *lpxT*-HindIII | ATGAAAGCTTGGTGCGCATCATCAGGATTATCCTCA |

^a^The restriction sites used for insertion of amplicons in the expression vectors are indicated in the primer’s names and it are underlined in the sequences.

**Table S4.** Primers used for Q-PCR

| arnT-1 | TATTGCATTCTGGCTGGTGG |
| --- | --- |
| arnT-2 | TTTTTGCGTCGCTACCCATG |
| wzzEC-1 | ACAGCAATTATCACTCAGCC |
| wzzEC-2 | GTTTTTCTCGTTCTTCCTGA |
| wzzfepE-1 | CTGGCTCAGGATCGCATTAA |
| wzzfepE-2 | TCTGCACCGAGAGAAATAGA |
| rrsA | CAGCCACACTGGAACTGAGA |
| rrsA | CCGAAGGCCTTTTTCATACA |
| gyrA | TGAACGGTTCTTCCGGTATC |
| gyrA | ATGTGTTCCATCAGCCCTTC |
| ffh | GCGCTAAGCCAGAAATCATC |
| ffh | ATTCCGCCCTTCTTCATTTT |
